# Seasonal Pulses of Marburg Virus Circulation in Juvenile *Rousettus aegyptiacus* Bats Coincide with Periods of Increased Risk of Human Infection

**DOI:** 10.1371/journal.ppat.1002877

**Published:** 2012-10-04

**Authors:** Brian R. Amman, Serena A. Carroll, Zachary D. Reed, Tara K. Sealy, Stephen Balinandi, Robert Swanepoel, Alan Kemp, Bobbie Rae Erickson, James A. Comer, Shelley Campbell, Deborah L. Cannon, Marina L. Khristova, Patrick Atimnedi, Christopher D. Paddock, Rebekah J. Kent Crockett, Timothy D. Flietstra, Kelly L. Warfield, Robert Unfer, Edward Katongole-Mbidde, Robert Downing, Jordan W. Tappero, Sherif R. Zaki, Pierre E. Rollin, Thomas G. Ksiazek, Stuart T. Nichol, Jonathan S. Towner

**Affiliations:** 1 Viral Special Pathogens Branch, Centers for Disease Control and Prevention, Atlanta, Georgia, United States of America; 2 National Institute of Communicable Diseases, Special Pathogens Unit, Johannesburg, South Africa; 3 Biotechnology Core Facility Branch, Centers for Disease Control and Prevention, Atlanta, Georgia, United States of America; 4 Uganda Wildlife Authority, Kampala, Republic of Uganda; 5 Infectious Disease Pathology Branch, Centers for Disease Control and Prevention, Atlanta, Georgia, United States of America; 6 Division of Vector-borne Diseases, Arbovirus Diseases Branch, Centers for Disease Control and Prevention, Atlanta, Georgia, United States of America; 7 Integrated BioTherapeutics, Gaithersburg, Maryland, United States of America; 8 Uganda Virus Research Institute, Entebbe, Republic of Uganda; 9 Global AIDS Program, Centers for Disease Control and Prevention, Atlanta, Georgia, United States of America; University of Wisconsin-Madison, United States of America

## Abstract

Marburg virus (family *Filoviridae*) causes sporadic outbreaks of severe hemorrhagic disease in sub-Saharan Africa. Bats have been implicated as likely natural reservoir hosts based most recently on an investigation of cases among miners infected in 2007 at the Kitaka mine, Uganda, which contained a large population of Marburg virus-infected *Rousettus aegyptiacus* fruit bats. Described here is an ecologic investigation of Python Cave, Uganda, where an American and a Dutch tourist acquired Marburg virus infection in December 2007 and July 2008. More than 40,000 *R. aegyptiacus* were found in the cave and were the sole bat species present. Between August 2008 and November 2009, 1,622 bats were captured and tested for Marburg virus. Q-RT-PCR analysis of bat liver/spleen tissues indicated ∼2.5% of the bats were actively infected, seven of which yielded Marburg virus isolates. Moreover, Q-RT-PCR-positive lung, kidney, colon and reproductive tissues were found, consistent with potential for oral, urine, fecal or sexual transmission. The combined data for *R. aegyptiacus* tested from Python Cave and Kitaka mine indicate low level horizontal transmission throughout the year. However, Q-RT-PCR data show distinct pulses of virus infection in older juvenile bats (∼six months of age) that temporarily coincide with the peak twice-yearly birthing seasons. Retrospective analysis of historical human infections suspected to have been the result of discrete spillover events directly from nature found 83% (54/65) events occurred during these seasonal pulses in virus circulation, perhaps demonstrating periods of increased risk of human infection. The discovery of two tags at Python Cave from bats marked at Kitaka mine, together with the close genetic linkages evident between viruses detected in geographically distant locations, are consistent with *R. aegyptiacus* bats existing as a large meta-population with associated virus circulation over broad geographic ranges. These findings provide a basis for developing Marburg hemorrhagic fever risk reduction strategies.

## Author Summary

Marburg virus, like its close relative Ebola virus, can cause large outbreaks of hemorrhagic fever with case fatalities nearing 90%. For decades the identity of the natural reservoir was unknown. However, in 2007 Marburg viruses were isolated directly from Egyptian fruit bats (*Rousettus aegyptiacus*) that inhabited a Ugandan gold mine where miners were previously infected. Soon after, two tourists became infected with Marburg virus after visiting nearby Python Cave, a popular attraction in Queen Elizabeth National Park, Uganda. This cave also contained *R. aegyptiacus* bats (∼40,000 animals). These events prompted a long-term investigation of Python Cave to determine if, 1) *R. aegyptiacus* in the cave carried infectious Marburg virus genetically similar to that found in the tourists, and 2) what ecological factors might influence virus spillover to humans. In the study, we found that, 1) approximately 2.5% of the bat colony is actively infected at any one time and that virus isolates from bats are genetically similar to those from infected tourists, and 2) specific age groups of bats (juveniles∼six months of age) are particularly likely to be infected at specific times of the year that roughly coincide with historical dates of Marburg virus spillover into humans.

## Introduction

Marburg virus (family *Filoviridae*), is the etiologic agent of Marburg hemorrhagic fever (MHF), a severe disease associated with person-to-person transmission and high case fatality. The virus was discovered in August 1967 when simultaneous outbreaks of MHF occurred in laboratory workers in Germany and Yugoslavia [1], [2]. The source of the virus was associated with importation of infected African green monkeys (Cercopithecidae: formerly *Cercopithecus aethiops*; currently *Chlorocebus tantalus* [3]) consigned from Uganda to Europe for use in the laboratories where the outbreaks occurred [4].

Since its discovery, the sporadic nature of Marburg virus outbreaks and the diverse history of human exposures have made it difficult to definitively trace the virus to its natural source, but mounting evidence has shown a recurrent link to caves or mines, leading investigators to suspect bats as a likely reservoir. In early February 1975, the second known outbreak of MHF occurred after two tourists traveled through Zimbabwe and reported sleeping in rooms with bats and visiting Chinhoyi caves in the days before developing symptoms [5]. In January 1980, and then again in August 1987, two patients contracted MHF after visiting a cave complex with large bat populations on Mt Elgon, Kenya. From 1998–2000, a protracted outbreak occurred at the Goroumbwa mine in Durba village in northeast Democratic Republic of Congo (DRC) and consisted of multiple short chains of virus transmission among gold miners and their families [6]. A concomitant ecological investigation found the mine to be populated with large numbers of bats of several species, three of which were later found to have evidence of Marburg virus infection, most notably the Egyptian fruit bat *Rousettus aegyptiacus* (order Chiroptera: family Pteropodidae) which had the highest prevalence (20.5%) of antibody to the virus [7]. In 2005, a healthcare center-based outbreak in Uige, northern Angola, became the first MHF outbreak to be detected on the west coast of Africa and the largest MHF outbreak on record [8]. The origin of the Angola outbreak was never determined, but that same year in nearby Gabon, a survey of 1,100 bats representing 10 bat species found only the cave-dwelling *R. aegyptiacus* to be positive for evidence of Marburg virus infection [9]. However, in both the Gabon and Durba DRC studies, scientists were unable to isolate Marburg virus from infected bat tissues.

In July and September 2007, MHF re-emerged in gold miners, this time in southwest Uganda at the Kitaka mine which is approximately 1,280 km from Durba. Here, genetic evidence showed two independent virus introductions from the natural reservoir into humans. A mark-recapture study estimated the mine to populated by over 100,000 *R. aegyptiacus*, from which five genetically diverse Marburg virus isolates were obtained from bats collected over an eight month period, demonstrating that *R. aegyptiacus* can naturally harbor infectious Marburg virus and that multiple lineages of virus can persist in a same bat colony for an extended period [10].

A year later, in late June 2008, MHF again occurred in southwest Uganda. This case involved a Dutch tourist who became fatally infected following a visit to Python Cave in Queen Elizabeth National Park (QENP) [11]. Python Cave is a popular tourist attraction 50 linear kilometers from the Kitaka mine and is known for the large African rock pythons that give the cave its name, but more importantly, its large *R. aegyptiacus* colony upon which the snakes feed. The publicity from the Dutch MHF case resulted in the retrospective identification of a second, non-lethal, MHF case associated with Python Cave. This individual was an American tourist who visited the bat colony in late December 2007 and developed MHF symptoms soon after returning home to Colorado, USA [12].

Together, these epidemiologic and laboratory data indicate *R. aegyptiacus* is a natural reservoir for Marburg virus. However, important questions remain such as how the virus naturally persists in these bats, and what ecological drivers cause occasional spillover from bats to humans. In the present study, we report a multi-year investigation of natural Marburg virus circulation among *R. aegyptiacus* in southwest Uganda, with emphasis on bats inhabiting Python Cave. Our data show a dynamic pattern of Marburg virus transmission that produces cyclical fluctuations in active infections associated with defined age cohorts of the bat population.

## Results/Discussion

### Description of Python Cave and bat collections

In response to the infection of the American and Dutch tourists, a series of four ecological investigations were conducted at Python Cave from August 2008 through November 2009. The goals of this study were to 1) determine if Marburg virus infected bats were present in the cave, and if so, what species of bat; and 2) determine what ecological factors, if any, may have led to the human infections. *Rousettus aegyptiacus* breed twice a year, becoming pregnant around November and May and giving birth in February and August, respectively (gestation period is approximately 105–107 days based on captive observations) [13]. The bat collections were scheduled during peak breeding or birthing periods (August 2008, February 2009, August 2009, November 2009) and were designed to complement two previous studies at the nearby Kitaka mine which were also carried out during similar peak times of either the birthing or breeding seasons (August 2007 and May 2008 respectively). Based on comparisons to the Kitaka mine, which contained over 100,000 *R. aegyptiacus* and a large number of smaller insectivorous bats (*Hipposiderous spp.*), the bat population at Python Cave was estimated to be at least 40,000 animals, and *R. aegyptiacus* was the sole chiropteran inhabitant of the cave.

Python Cave is actually a tunnel open at both ends, and is approximately 15 meters (m) long and 12 m wide, formed by a subterranean stream that undercut a land bridge spanning a small gorge. The height of the interior is variable, ranging from 3.5 m to nearly 5 m due to the boulder strewn floor, and the cave contains numerous nooks, crevices and hidden chambers, with nearly every square centimeter of ‘hanging space’ used by the bats. The limited space forces bats to occupy sunlit ledges of the gorge on either side of the tunnel openings. Most juvenile bats were observed roosting in these more peripherally located pockets and ledges near the ground, both inside and outside of the tunnel proper while adults tended to occupy the darker interior. These juvenile bats were also observed roosting on the sides of the larger boulders and in holes on the cave floor.

In addition to the bats, other vertebrate fauna observed in the cave included at least two large African rock pythons (*Python sebae*), and several forest cobras (*Naja melanoleuca*). Also observed visiting the cave were African fish eagles (*Haliaeetus vocifer*), palm-nut vultures (*Gypohierax angolensis*), Nile monitor lizards (*Varanus niloticus*) and olive baboons (*Papio anubis*). Further, a variety of invertebrates were found, most notably argasid ticks (Family Argasidae) on the cave walls, nycteribiid flies (Family Nycteribiidae) in the bat pelage, and fresh water crabs (Crustacea: Decapoda) in the subterranean stream beneath the cave floor.

Over the four sampling periods at Python Cave, 1,622 *R. aegyptiacus* were captured and tested for Marburg virus. Both genders were represented nearly equally (Table 1). Of the 798 females captured, 449 were of active breeding age evidenced by having an attached pup, being pregnant or having enlarged nipples indicative of previous lactation. Of the 824 males captured, 453 were scrotal. The majority (61%) of the total captures (n = 1,622) were adults (n = 993; forearm length >89 mm) while the remainder consisted of volant juveniles (n = 417) or newborn pups (n = 212).

**Table 1:** Summary of *Rousettus aegyptiacus* caught at Python Cave displayed by class, and PCR, virus isolation, and ELISA results.

		Captures	PCR +	Isolates	Ab +
**Female**	Adult	499	4	2	139
	Non-adult	299	17	2	20
	**Total**	**798**	**21**	**4**	**159**
**Male**	Adult	494	7	—	75
	Non-adult	330	12	3	16
	**Total**	**824**	**19**	**3**	**91**
**Total**		**1622**	**40**	**7**	**250**

### Evidence of Marburg virus infection by Q-RT-PCR and virus isolation from bat tissues

Viral RNA extracted from pooled liver and spleen samples were tested for Marburg virus RNA using a real-time Q-RT-PCR assay designed to detect all known strains of Marburg virus [10]. Of the 1,622 bats captured, 40 (2.5%) were actively infected as evidenced by having detectable Marburg virus RNA (Q-RT-PCR positive). A population estimate of 40,000 bats combined with an infection level of 2.5% estimates approximately 1,000 actively infected bats to reside inside this popular tourist destination at certain times of the year. Several other tissues tested positive for Marburg virus RNA (Table 2) and always in conjunction with positive liver and spleen samples, including kidney (*n* = 2), colon and rectum (*n* = 5), lung (*n* = 8), heart (*n* = 3), intestine (*n* = 3) and blood (*n* = 2). The array of virus-infected tissues indicates that *R. aegyptiacus* inhabiting Python Cave are probably in diverse stages of infection. Some bats, (e.g. bat #843 in Table 2) appear acutely and systemically infected as evidenced by simultaneous infection of lung, liver/spleen, kidney, colon, mid-gut, heart and blood. The Marburg virus-specific RNA loads found in blood of bats #843 and #1175 were very low (Ct values between 30–39; indicating lower amounts of viral RNA) and could not explain the higher RNA levels seen in the other infected tissues (Ct values between 20–30; indicating higher amounts of viral RNA). All bats with multiple Marburg virus-positive tissues were also positive by testing of pooled liver/spleen suggesting that liver and spleen remain the best target tissues for identifying Marburg virus-infected *R. aegyptiacus*. Finding Marburg virus in tissues from lung, kidney, colon, and mid-gut raises the possibility of virus shedding through an oral, fecal, or urinary route(s). One bat had Marburg virus-positive reproductive tissue (uterus/ovary) which, given the previous discovery of Ebola virus in reproductive tissue of infected humans [14]–[16] and active Marburg virus transmission via semen [17], raises the possibility of sexual transmission among bats. The potential involvement of arthropod vectors has not been ruled out, although limited numbers of argasid ticks (14 pools of 10–20 ticks) collected thus far from the cave were negative for Marburg virus RNA by Q-RT-PCR.

**Table 2:** Summary of *Rousettus aegyptiacus* found positive for Marburg virus in multiple tissues by Q-RT-PCR.

Date	Bat #	Sex	Age	Li/Sp	Heart	Lung	Kidney	Colon	Repro	Intestine*	Blood
Aug 09	843	Male	J	++++	+	++++	++	++	−	+++	++
Aug 09	849	Female	J	+	−	−	−	+	−	++	−
Aug 09	907	Female	J	+	−	−	−	−	−	+	−
Aug 09	914	Female	J	++	−	+	+	−	−	−	−
Aug 09	934	Female	J	+	−	−	−	++	−	−	−
Aug 09	960	Male	J	+	−	++	−	+++	−	−	−
Aug 09	1134	Female	J	+	−	+	−	−	−	−	−
Aug 09	1175	Male	J	+++	−	−	−	−	−	−	+
Nov 09	1232	Female	J	++	+	+	−	−	−	−	−
Nov 09	1261	Male	A	++	−	+	−	−	−	−	−
Nov 09	1304	Female	J	+++	++	++	−	+	+	−	−
Nov 09	1368	Male	J	++	−	+	−	−	−	−	−

For reference, approximate TCID50 values for positive tissues were derived from a standard curve of diluted stock virus (371Bat Uga 2007) assayed using the identical Q-RT-PCR assay as that used for the tissues.

J = juvenile bat (non-pup; forearm length ≤89 mm).

A = adult bat (forearm length >89 mm).

++++ = Ct 20–25 = (50,000–1,500,000 TCID_50_/ml).

+++ = Ct 25–30 = (2000–50,000 TCID_50_/ml).

++ = Ct 30–35 = (100–2000 TCID_50_/ml).

+ = Ct 35–39 = (5–100 TCID_50_/ml).

* Pool of 3 tissue sections.

From the Q-RT- PCR positive bats at Python Cave, seven genetically distinct Marburg virus isolates (Table 1) were obtained directly from homogenized liver/spleen tissue, and for one bat (#843) virus was additionally isolated from lung and blood (viremia). These virus isolates, combined with those from five bats captured at the Kitaka mine, bring to 12 the total number of bats from which Marburg virus has been isolated. In fact, Marburg virus was isolated at least once from each *R. aegyptiacus* collection expedition in Uganda, including those at the Kitaka mine [10], with the exception of the 2009 February/March Python Cave collection, which yielded no virus isolate. There were no significant differences in the ability to isolate virus from either Q-RT-PCR positive adults (2/11, 18.18%) or juveniles (5/28, 17.85%; *t* = −0.023, p>.98), or likewise, from males (3/19, 15.79%) or females (4/20, 20.0%; *t* = .334, p>.70). Successful isolation of Marburg virus roughly correlated with samples that had Ct values of 30 or less (>2000 TCID_50_/ml).

### Immunohistochemical analyses

Immunohistochemical analysis (IHC) was performed on formalin fixed liver and spleen tissues from all Q-RT-PCR positive bats and an approximate equal number of negative bats. Of the 40 Marburg virus positive bats, four (10%) were positive via IHC in liver, one of which (Bat #843) was additionally positive in spleen. All Q-RT-PCR positive heart, lung, kidney, colon and mid-gut tissues shown in Table 2 with Ct values less than 35 (virus loads >∼100 TCID_50_/ml), were additionally tested by IHC, but none were positive for Marburg virus antigen. There was no evidence of any pathology apparent during necropsies or IHC analysis that could be attributed directly to infection with Marburg virus. Moreover, there were no signs of overt morbidity or mortality witnessed during the capture or processing of the bats, including those actively infected with Marburg virus. However, the cave environment is such that dead or dying bats might not be visible for long periods of time due to predation, guano accumulation, and the large detritivore community living in the cave.

### Phylogenetic relationship of Marburg virus sequences from bats and humans and evidence of long distance *R. aegyptiacus* movement

**Figure 1:**
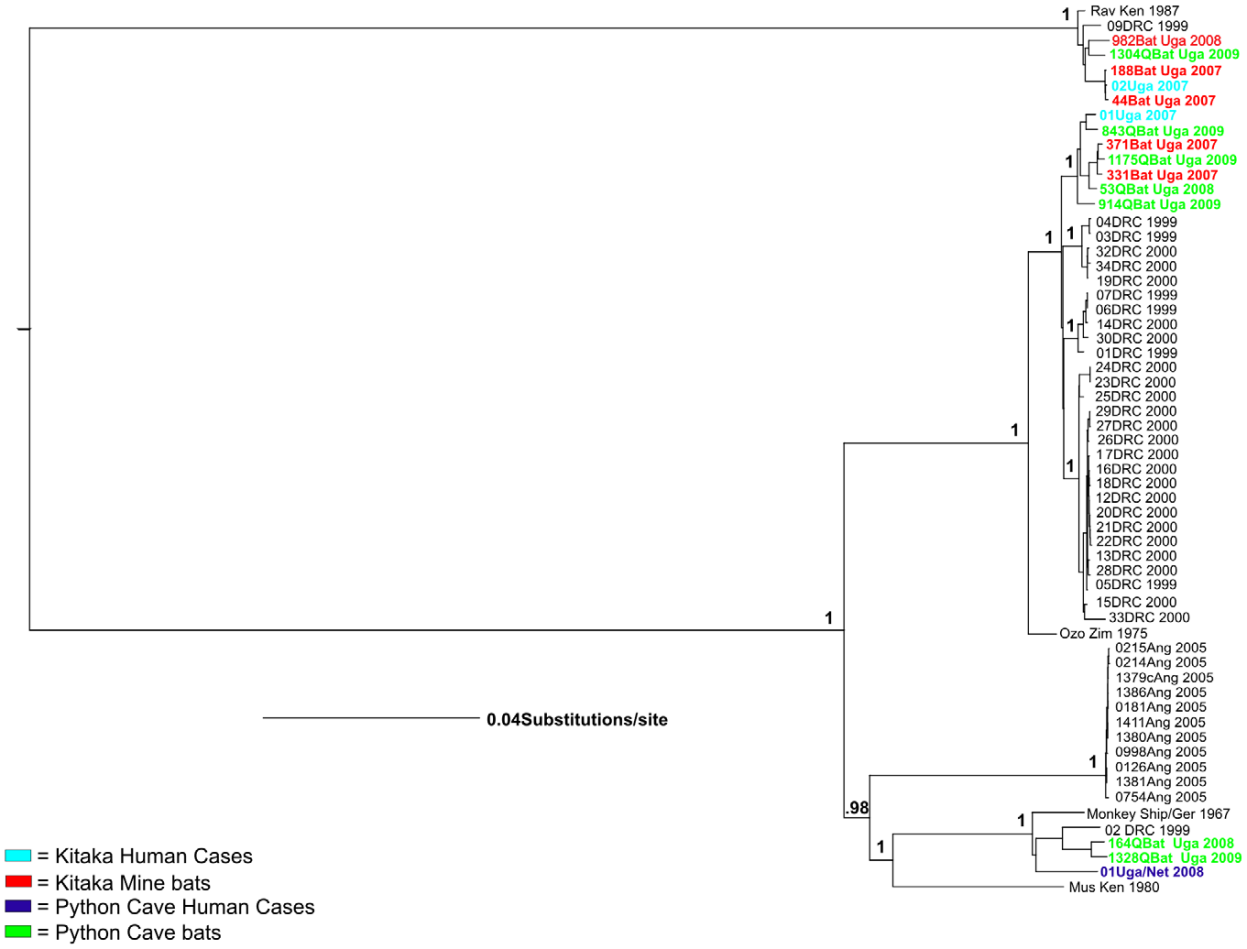
Bayesian phylogeny of full length Marburg genome. Phylogenetic results from a Bayesian analysis on full-length Marburg virus genome sequences from 12 Marburg bat isolates, 3 recent Ugandan human isolates from the two Kitaka miners (01Uga 2007, 02Uga 2007), and the Dutch tourist (01Uga/Net 2008), as well as 45 historical isolates (Table S2 for GenBank accession numbers). Posterior probabilities above .50 are shown above the appropriate nodes. Marburg virus sequences from human cases from Kitaka mine (Uganda 2007) in are in orange, sequences from human cases from Python Cave (2008 Uganda) are in blue, sequences from Kitaka Mine bats are in red, and sequences from Python Cave bats are in green.

**Figure 2:**
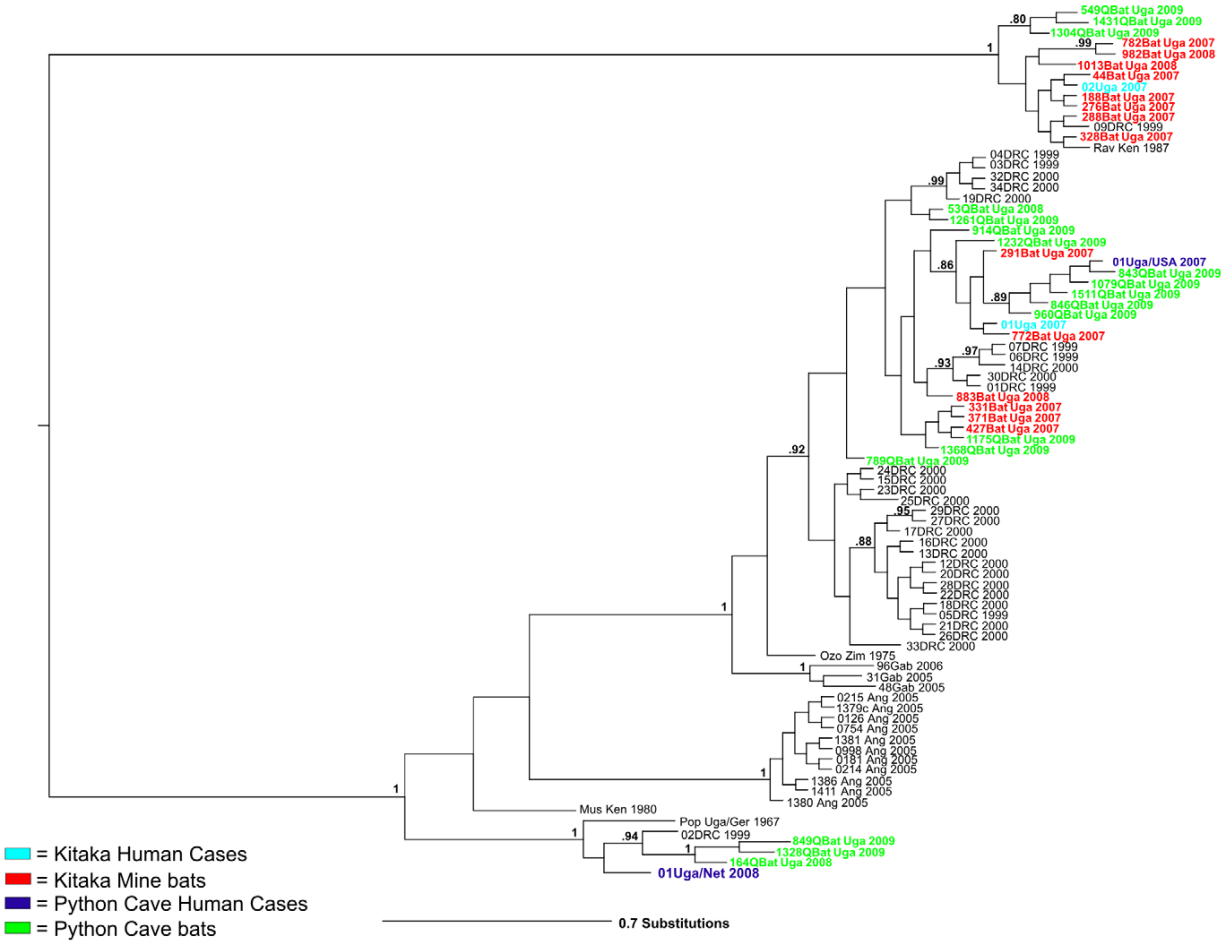
Bayesian phylogeny of Marburg NP and VP35 genes. Phylogenetic results from a Bayesian analysis on concatenated NP and VP35 sequence fragments obtained from bat specimens, historical isolates (45), and the recent Ugandan human samples (01Uga 2007, 02Uga 2007, 01Uga/Net 2008) as well as the American tourist (01Uga/USA 2007), for which there was no isolate, only partial Marburg virus sequence (Table S2 for GenBank accession numbers). Sequences 846QBat_Uga_2009, 849QBat_Uga_2009, 1079QBat_Uga_2009, 1261QBat_Uga_2009, 1328QBat_Uga_2009, and 1511QBat_Uga_2009 represent NP only. Posterior probabilities above .50 are shown above the appropriate nodes. Marburg virus sequences from human cases from Kitaka mine (Uganda 2007) in are in orange, sequences from human cases from Python Cave (2008 Uganda) are in blue, sequences from Kitaka Mine bats are in red, and sequences from Python Cave bats are in green.

Full-length genome sequences (19,114 bp) were determined from all seven of the Python Cave Marburg virus bat isolates. Two isolates (164QBat Uga 2008 and 1328QBat Uga 2009) closely match the sequence of the virus isolate obtained from the Dutch MHF case (01Uga/Net 2008; Fig. 1) based on a Bayesian analysis. Unfortunately, no virus was isolated from the American tourist, but the sequence from small portions of the NP and VP35 genes were obtained from clinical material following amplification by nested RT-PCR. The sequences were concatenated into a single ∼700 nt sequence and analyzed with corresponding Marburg virus sequences from bats and humans using similar Bayesian methods. As expected, multiple Marburg virus sequences from Python Cave bats closely match that of the American tourist (Fig. 2). Further, these two analyses produced phylogenies showing that the entire known genetic spectrum of Marburg virus, >20% nucleotide diversity, can be found circulating in Python Cave at any one time. This finding is consistent with *R. aegyptiacus* representing a *bona fide* long term reservoir species for the virus.

The fact that several of the Marburg virus sequences from Python Cave and Kitaka mine are similar to sequences obtained from distant regions of sub-Saharan Africa including Gabon (48Gab 2005, 31Gab 2005, and 96Gab 2006) and Zimbabwe (OzoZim 1975) suggest that there is considerable animal movement over long distances and exchange of infectious virus through a network of *R. aegyptiacus* colonies that span the continent. As proof of direct animal movement between *R. aegyptiacus* bat colonies, a numbered collar was found at Python Cave in August 2008 that had been initially placed on an adult female *R. aegyptiacus* bat at the Kitaka mine during the mark and recapture study three months earlier [10]. The Kitaka mine and Python Cave are separated by roughly 50 linear kilometers and separated by tracts of dense forest and zones of agricultural activity. In South Africa, marked *R. aegyptiacus* have been shown to move up to 32 km between roosting sites and in one instance, a marked female relocated to a site 500 km away [18]. Additional evidence of direct movement between colonies was found when a second *R. aegyptiacus* bat, marked as a male juvenile at the Kitaka mine in 2008, was captured at the Python Cave as an adult in August of 2009, a full 15 months after the initial capture and marking.

### Older juvenile bats are most likely to be actively infected with Marburg virus

In the initial 2007 Kitaka mine investigation [10], a significantly higher proportion of juvenile bats were found to be actively infected than were adults (12% vs 4.2% respectively), yet in the follow-up study at the same location nine months later (in May 2008), the proportions of infected juveniles and adults were slightly inverted (1.7% vs 5.7% respectively) [10]. From these early data, it was hypothesized that perhaps the reason for the difference in infection prevalence resided in factors related to the age of the juvenile cohorts, being six months old during the birthing seasons (August and February) yet only three months old during the breeding seasons (May and November). At the time of capture, older juveniles (six months old) would have been weaned for at least four months, fully independent and without any residual Marburg-specific maternal antibody if they were born to an antibody positive mother. In contrast juveniles caught during breeding seasons (May and November) would be roughly three months old, barely independent, and newly released from the physically occlusive protection of their mother. Newborn pups remain attached to the nipple and well under the wing of the mother for the first six weeks of their lives and then remain in close contact, occasionally clinging to the mother's back for an additional two weeks (Towner and Amman personal observations of captive *R. aegyptiacus* bats).

**Figure 3:**
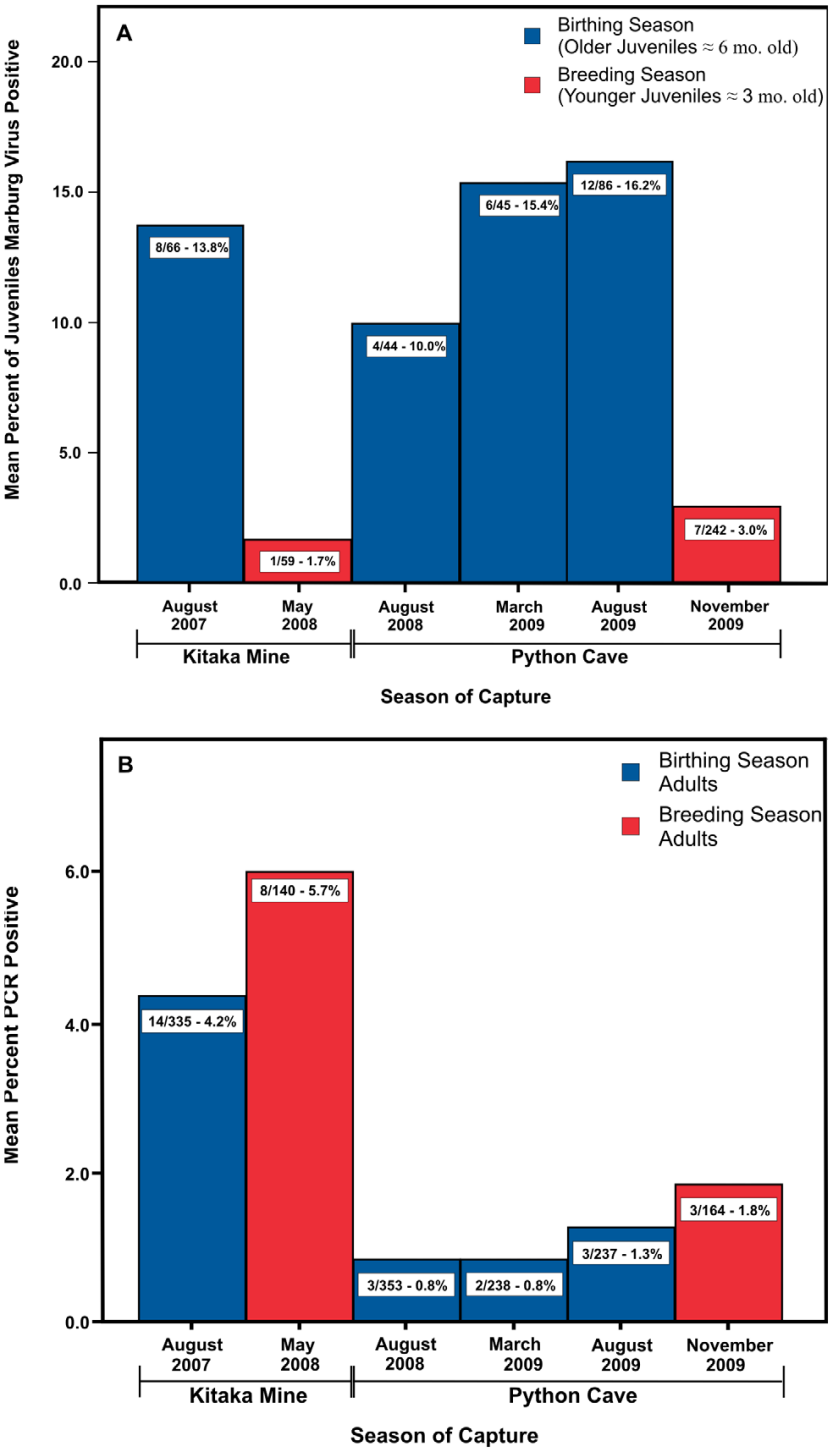
Percent active infection among older and younger juvenile bats and adults. (A) Histogram showing the percent of juvenile bats from Kitaka Mine and Python Cave actively infected (Q-RT-PCR+) with Marburg virus during breeding and birthing seasons. (B) Histogram of the percent of adult bats from Kitaka Mine and Python Cave actively infected (Q-RT-PCR+) with Marburg virus during breeding and birthing seasons.

Analysis of the Python Cave Q-RT-PCR data reveals a seasonal age bias among Marburg virus-infected bats which correlates with that observed at Kitaka mine [10]. Of the 40 total Q-RT-PCR positive bats from Python Cave, 29 (of 627 total) were juveniles compared to 11 (of 994 total) adults (*t* = 3.898, p<.001). When the active infection data from the Kitaka mine and Python Cave investigations are combined and sorted into three age categories, young juveniles, old juveniles and adults, a reproducible age-linked infection pattern emerges (Fig. 3a). Levels of active infection among young juveniles remain around 2–3% (8/301, 2.65%) and increase to 10–15% by six months of age (30/241, 12.4%; *t* = −4.212, p<.001). Adults by contrast maintain a relatively constant level of active infection (Fig. 3b) ranging from 2–5% (33/1467, 2.4%), irrespective of season (breeding season = 11/305, 3.6%; and birthing season = 22/1163, 1.9%; *t* = 1.508, p>.13%). Interestingly, no evidence of vertical transmission was found. In one instance, a Q-RT-PCR positive mother was identified with an Q-RT-PCR negative pup. Moreover, all pups from either Kitaka mine or Python Cave (n = 223) tested uniformly negative for active Marburg virus infection.

Together, these data present a dynamic picture of natural Marburg virus circulation in which juveniles are exposed to the virus at an early stage of their development following independence at three months of age and increasing up through their first six months of life. Once in the adult population after seven to eight months of age, the incidence of infection apparently drops off for reasons not currently understood and levels out to a more constant rate that is independent of season. We are currently developing reliable measures for sub-adult age classification, but until they are complete, tracking the younger age cohorts beyond six to seven months of age remains difficult.

The overall pattern of horizontal transmission is supported by serological data from the Python Cave bats in which Marburg virus-specific IgG antibody prevalence increases with age starting from 4.1% (10/242) among young juveniles and increases to 14.8% (26/175) among older juveniles and finally reaches 21.5% (214/993) in adults. The lower infection levels observed in young juveniles is likely due to lack of physical opportunity for exposure to other members of the population perhaps aided by maternal antibody protection for those pups born to antibody positive mothers. In our analyses, all pups of antibody positive mothers (*n* = 20) were themselves antibody positive. It is unknown if maternal antibody is actually protective.

We speculate that the introduction of Marburg virus into the juvenile bat population may also be influenced by the positioning of bat groups within the cave. On every occasion, segregation of juveniles (non-pups) from adults was witnessed with juvenile bats generally pushed to the periphery of the cave away from the center where it is darkest. At the periphery, juveniles were observed roosting tightly together primarily in small holes or on the sides of large boulders on the cave floor. Occasionally small groups of juveniles could be found low on the walls but outside the cave in filtered sunlight. The cave floor contains copious amounts of accumulated guano (feces and urine) that are continually refreshed by new deposits. Should virus be shed through bat excretions, the physical positioning of juvenile bats directly underneath the adult bats would make juvenile bats particularly susceptible to virus exposure. Unfortunately, testing of limited (<100 samples) urine and fecal samples for viral RNA has not yet yielded positive results, probably due to persistent Q-RT-PCR inhibitors that have thus far hindered our ability to detect Marburg virus RNA in experimentally spiked guano samples in the laboratory (data not shown). Nevertheless, finding of Marburg virus-positive kidney, colon/rectum, and intestine samples, suggests virus shedding through excreta may well occur.

As the juveniles age and are recruited into the adult population or disperse to other caves or suitable sites, the low lying roosting areas are repopulated by the next pulse of newly weaned juveniles. These juveniles in-turn become infected, spreading the virus primarily amongst themselves until they too disperse or move into the adult population. This cycle continues season after season to perpetuate virus transmission within the colony. The pattern of continual circulation of the virus within the population coupled with the continued lack of any overt morbidity and mortality in infected bats is consistent with expectations for *Rousettus aegyptiacus* being a natural reservoir for Marburg virus.

### Seasonal clustering of spillover events to humans coincide with peaks of infection in juvenile bats

**Figure 4:**
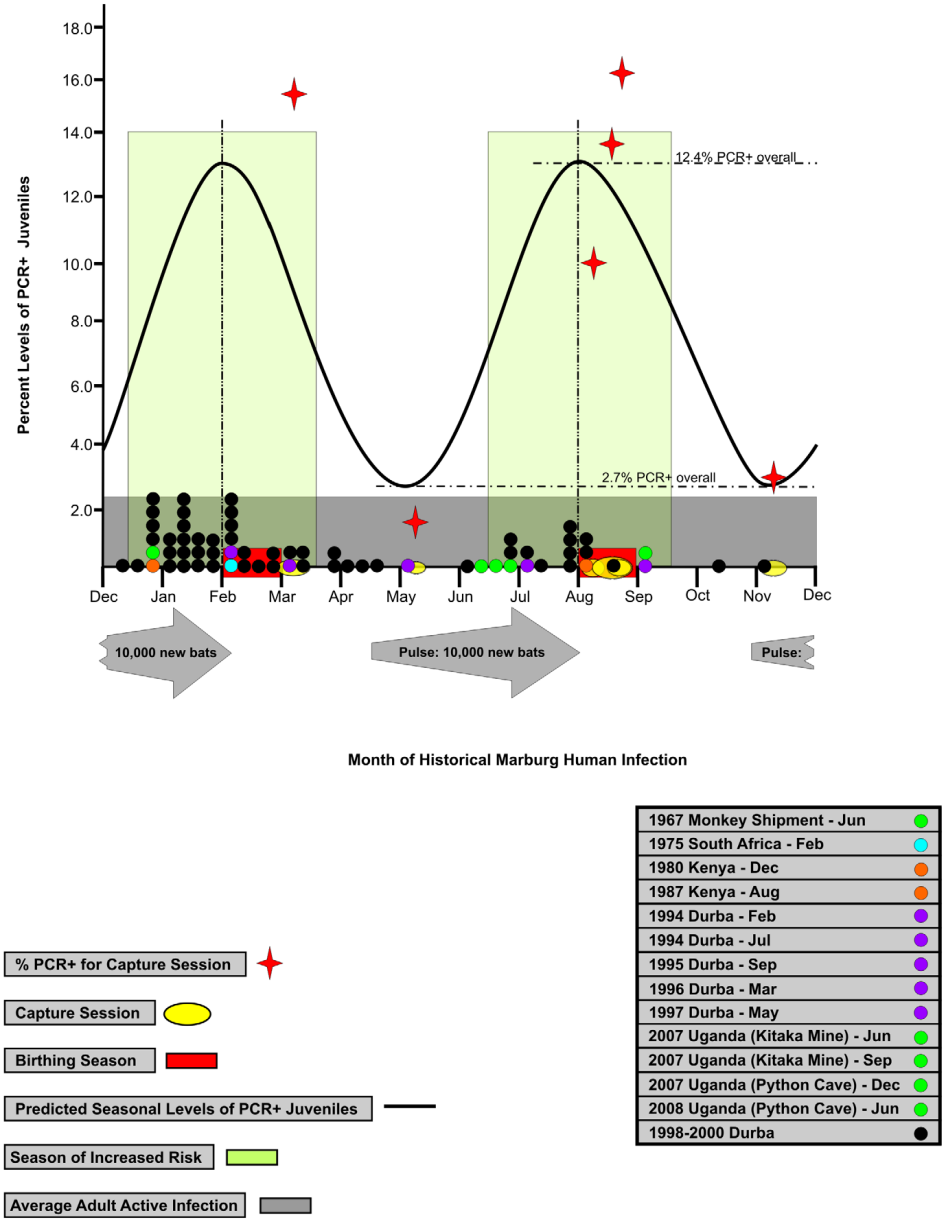
Increases in seasonal risk to human health. Historical spillover events (colored circles on X axis) compared to predicted seasonal levels of PCR+ juveniles (sinusoidal curve). The amplitude of the curve is based on average PCR+ juveniles experimentally determined during birthing (12.4%) and breeding (2.7%) seasons. Large light green vertical rectangles represent the proposed approximate three month seasons of increased risk based on the average level of juvenile infected bats at peak times of encompassing birthing (February and August) and breeding (May and November). Large gray arrows depict the twice yearly influx of newly autonomous juvenile bats born in the prior birthing season. The influx begins at the approximate time of the juvenile's independence from their mothers.

The approximate dates of 13 suspected Marburg virus spillover events were determined from the literature (Table 3), seven of which were linked directly to subterranean gold mining activities at the bat-inhabited mines in Durba, DRC from 1994–1997 [6] and Ibanda, Uganda 2007 [10]. Five spillover events involved tourists with defined dates of visitation to caves containing *R. aegyptiacus*, in the weeks just before the onset of MHF symptoms. The original 1967 outbreak was also included, and for that, a date was chosen that was one incubation period (three weeks) prior to the first shipment of infected monkeys that arrived in Frankfurt, Germany on 21 July 1967 (via London Heathrow airport) and further distributed within Germany (Marburg and Frankfurt) and to Belgrade, Yugoslavia [19]. When all 13 Marburg virus spillover events are listed by month of occurrence, the data show a temporal clustering of human infections, coinciding with the summer (mid-June through mid-September) and winter months (mid-December through mid-March) of the northern hemisphere. The majority of spillover events (7/13) involved resident African miners, suggesting that the clustering effect was not due to seasonal tourism. More importantly, when the dates of these 13 spillover events are compared to a sinusoidal curve derived from the field collection data showing the seasonal incidence of juvenile *R. aegyptiacus* infections (Fig. 4), a pattern of coincidence emerges. The sinusoidal curve has peaks and troughs that correspond to the beginning of the birthing and breeding seasons respectively, each separated by roughly three months, and whose peak heights reflect the average percentage of infected juveniles for each seasonal category. These data show that 11 of 13 (84.6%, Fisher's Exact Test p<.05) spillover events occurred during the three month periods encompassing each of the two biannual birthing seasons when juvenile bats are roughly 4.5–7.5 months old and most likely to be infected with Marburg virus. Moreover, when suspected (extrapolated) exposure dates for 52 primary cases (all miners and epidemiologically unlinked to any other human cases; Table S1) from the final MHF patient list from the 1998–2000 outbreak in Durba, DRC [6] are included in the analysis (Pierre Rollin and Robert Swanepoel; personal communication; Table S2), 54 of 65 (83.1% Fisher's Exact Test p<.05) spillover events occur during the same periods encompassing each of the biannual birthing seasons, further supporting the idea that these three-month periods may represent times of increased risk for exposure to Marburg virus. The contribution of young naïve bats to the overall population during these seasons is considerable. Based on a population estimate of 40,000 bats in Python Cave and 80% pregnancy of sexually active females [10], [20], the number of births at Python Cave could easily exceed 20,000 pups a year (10,000 pups every 6 months). Many of those pups will become juveniles that are ultimately pushed to the periphery of the cave where they may be more likely to encounter humans.

**Table 3:** Historical Marburg spillover events with dates of initial exposure excluding the 2005 Angola outbreak because the initial exposure date was never identified.

Date of Exposure	Country	Citation
30 Jun 1967	Germany Yugoslavia via Uganda	Extrapolated by subtracting one incubation period (21 days) from the date of the shipment received listed in [4], [19].
1–9 Feb 1975	South Africa via Zimbabwe	Index case traveled in Rhodesia Feb 1–9, admitted on 15 Feb 1975 [31].
25 Dec 1980	Kenya	Kitum (Elgon) Cave 25 December –15 days before illness [32].
1 Aug 1987	Kenya	Kitum Cave – 9 days before illness [33].
Feb 1994	DRC - Durba	Identified in Fig. 3 of Bauch et al [6].
Jul 1994	DRC - Durba	Identified in Fig. 3 of Bauch et al [6].
Sep 1995	DRC - Durba	Identified in Fig. 3 of Bauch et al [6].
Mar 1996	DRC - Durba	Identified in Fig. 3 of Bauch et al [6].
May 1997	DRC - Durba	Identified in Fig. 3 of Bauch et al [6].
10 June 2007	Uganda	Epidemiological data obtained during an outbreak investigation [34].
14 Sep 2007	Uganda	Epidemiological data obtained during an outbreak investigation [34].
25 Dec 2007	USA via Uganda	[12].
19 Jun 2008	Netherlands via Uganda	[11].

We conclude that Marburg virus transmission within the *R. aegyptiacus* colony occurs year round at a baseline level, and that the months surrounding the peak birthing seasons represent times of increased infection among juveniles. Further, the coincidence of peak periods of juvenile bat infections with the historical clustering of individual spillover events to humans at similar times of the year suggests these seasonal periods might represent periods of heightened public health risk perhaps due to the positioning of the juvenile roosting sites within the cave. These data provide the first long-term monitoring of any filovirus circulating in nature and provide a foundation for understanding ecological drivers that may instigate MHF outbreaks.

## Materials and Methods

### Bat capture and processing

All procedures listed herein (including those referred to in Towner et al. [10]), were performed in accordance with an institutionally approved animal care and use protocol (animal use protocol 1731AMMULX approved by the Centers for Disease Control and Prevention Institutional Animal Care and Use Committee). All aspects of the bat collections were undertaken with the approval of the Uganda Wildlife Authority and following the American Veterinary Medical Association guidelines on euthanasia and the National Research Council recommendations for the care and use of laboratory animals [21], [22].

Without exception, protective equipment (PPE) standard for working with filoviruses in the field setting was used [23]. Briefly, all personnel donned double latex gloves, disposable Tyvek suit, rubber boots, fitted p100 respirators (3M) and eye protection (in the form of a full face shield or full-face respirator) prior to entering the cave. When appropriate, personnel used bite-resistant gloves, full face shields, caving helmets for head protection, and due of the presence of multiple venomous snakes, Kevlar chaps to prevent snake bites on the lower extremities. All personnel were misted down with 3% Lysol immediately upon exit of the cave. During necropsies, PPE was less cumbersome but included double latex gloves, disposable gowns, and powered air-purifying respirator (PAPR) units (3M).

To maximize the chances of isolating virus, large numbers of *R. aegyptiacus* were sampled over the course of four separate collections spanning one year and three months beginning in August 2008. Bats were captured and processed following procedures detailed in Towner et al. [10]. The notable exceptions to those procedures were that harp traps were used exclusively to capture bats and more tissue types were collected. Replicate tissue samples were also preserved in 10% formalin for a minimum of four days and later changed to 70% ethanol for long term storage. Bats were identified morphometrically [24] and their measurements, sex, and breeding status were recorded

### Collection of additional fauna

Adult and nymphal argasid ticks (14 pools of 10–20) were collected from crevices in the rocks near bat roosting sites and immediately placed in chaotropic RNA extraction buffer. Collections of endoparasites occurred during necropsies and were identified as tongue worms of the phylum Pentastomida. These parasites were typically found on the liver and spleen.

### Virus isolation

Virus isolation attempts were carried out as described in Towner et al. [10]. Briefly, approximate 250 mg frozen tissue sections were placed on ice and homogenized in viral transport medium (HBSS/5% fetal calf serum) using sterile alundum (Fisher cat# A634-3) to form 10% suspensions. The homogenate was then spun at low speed for 5–10 minutes a 4°C and 100 ul of resulting supernatant was used to inoculate Vero E6 cells in 25 cm^2^ flasks at 37°C/5% CO_2_ for 1 hr. Media was then replaced with MEM/2% fetal calf serum and monitored for 14 days with a media change on day 7. All cultures were then tested by IFA for Marburg virus.

### Q-RT-PCR, RT-PCR and nucleotide sequencing analysis

Q-RT-PCR, RT-PCR, and nucleotide sequencing, were all performed using reagents and procedures described in Towner et al. [10]. Briefly, virus inactivation in tissue samples was achieved by incubating approximate 100 mg of tissue samples from bats in 450 µl of 2X cellular cold lysis buffer (ABI) at 4°C for greater than eight hours. Each tissue was then diluted to 1X and homogenized for 2 minutes, at 1500 strokes/min using a ball-mill tissue grinder (Genogrinder 2000, Spex Centriprep). Total RNA was extracted from 150 ul of the homogenate [25] and tested for Marburg virus using slightly modified Q-RT-PCR [8] or nested RT-PCR assays. The Q-RT-PCR assay consisted of two reporter probes, 5′ Fam-ATCCTAAACAGGC“T”TGTCTTCTCTGGGACTT-3′ and 5′ Fam-ATCCTGAATAAGC“T”CGTCTTCTCTGGGACTT-3′ in addition to the amplification primers (forward) 5′-GGACCACTGCTGGCCATATC-3′ and (reverse) 5′-GAGAACATITCGGCAGGAAG-3′. The quencher BHQ1 was placed internally in the probes at the “T” locations. The nested VP35 RT-PCR assay is previously described [6], and consisted of primers F1 (forward-outside) 5′-GCTTACTTAAATGAGCATGG-3′, F3 (forward-inside) 5′- CAAATCTTTCAGCTAAGG-3′, R1 (reverse-outside) 5′- AGIGCCCGIGTTTCACC-3′ and R2 (reverse-inside) 5′- TCAGATGAATAIACACAI ACCCA-3′. The four primers used for the nested NP assay [9] are MBG704F1 (forward-outside) 5′-GTAAAYTTGGTGACAGGTCATG-3′, MBG719F2 (forward-inside) 5′-GGTCATGATGCCTATGACAGTATCAT, MBG1248R1 (reverse outside) 5′- CTCGTTTCTGGCTGAGG-3′, and MBG1230R2 (reverse inside) 5′-ACGGCIAGTGTCTGACTGTGTG-3′. The annealing conditions were 50°C for the first round (both assays) and 54°C (NP assay) or 50°C (VP35 assay) for the second round using high-fidelity one-step RT-PCR reagents (Invitrogen). Primer concentrations and amplification conditions used were as described by the manufacturer. Sequencing was performed using the appropriate amplification primers and standard di-deoxy sequencing methods.

### Serology

Briefly, IgG detection was performed essentially as described in [26] with the exception that 96-well plates were coated with 200 ng/well of purified Marburg (Musoke) GP (Integrated BioTherapeutics, Gaithersburg, MD) or 200 ng/well of purified Ebola (Zaire) GP. The purified GPs contained a deletion of the trans-membrane domain (dTM) and were diluted in PBS. Bat sera were diluted 1∶100 and four-fold through 1∶6400 in 5% non-fat milk in PBS with 0.1% (vol/vol) Tween 20 (Bio-Rad Richmond, CA) and allowed to react with the GP-coated wells. Bound IgG was detected with goat anti-bat IgG (Bethyl cat# A140-118P) conjugated to horseradish peroxidase. Optical densities (OD) at 410 nm were recorded on a microplate spectrophotometer. The adjusted OD at 410 nm was generated by subtracting the OD of the well coated with Ebola-GP (dTM) from its corresponding Marburg GP-coated well. All sera were analyzed in duplicate and the threshold corrected ODs value for a positive Marburg IgG antibody test was determined to be 0.72 based on the mean corrected sum OD of the negative control group plus three standard deviations. The negative control group consisted of 210 young juvenile *R. aegyptiacus* (∼three months old). This age group was chosen because they were the cohort considered least likely to have evidence of previous Marburg infection based on data presented here and previously [10] that suggest Marburg virus is transmitted horizontally and not vertically between bats.

### Immunohistochemical analyses

Immunohistochemical analyses was performed following techniques described in [27] to determine if Marburg virus infection caused lesions in infected bats. Sections were cut from paraffin-embedded blocks prepared from formalin-fixed liver and spleen samples from 40 bats found positive by Q-RT-PCR, and examined concurrently with samples from 40 bats found negative by Q-RT-PCR. Hematoxylin and eosin (H&E) stained sections of the tissues were examined for lesions, and sections stained by an immune-alkaline phosphatase technique with a polyclonal rabbit anti-Marburg virus antiserum diluted to 1/1000.

### Statistical analysis

All statistical analyses, Fisher's Exact and two-sided independent samples T tests, of the capture data were performed using PASW 18.0 (SPSS Statistics, Rel. 18.0.0. 2009. Chicago: SPSS Inc. an IBM Company).

### Nucleotide sequencing and phylogenetic analysis

Sequencing of Marburg virus whole genomes and partial gene sequences (NP and VP35) were performed as previously described [8], [9]. Multiple sequence alignments were generated in SeaView [28] using the MAFFT function [29]. A Bayesian phylogenetic analysis was conducted in MrBayes 3.2 [30] using the GTR+I+G model of nucleotide substitution. Two simultaneous analyses, each with four Markov chains, were run for 10,000,000 generations, sampling every 100 generations. Convergence was examined prior to termination of the analysis by ensuring that the standard deviation of split frequencies had fallen below 0.01, thus confirming that the length of the run was sufficient. Trees generated before the stabilization of the likelihood scores were discarded (burnin = 100), and the remaining trees were used to construct a consensus tree. Nodal support was assessed by posterior probability values (≥.95 = statistical support). GenBank numbers for all sequences used in this study will be provided upon acceptance of this manuscript (see Table S2 for accession numbers).

## Supporting Information

Table S1 Suspected (extrapolated) exposure dates for 52 miners from the final Marburg hemorrhagic fever (MHF) patient list from the 1998–2000 outbreak in Durba, Democratic Republic of Congo.
(DOCX)

Table S2 GenBank accession numbers of all Marburg virus sequences analyzed.
(DOCX)
