# Supplementary material for: Seasonal Pulses of Marburg Virus Circulation in Juvenile Rousettus aegyptiacus Bats Coincide with Periods of Increased Risk of Human Infection
Source: PLoS Pathog. 2012 Oct 4;8(10):e1002877. doi: 10.1371/journal.ppat.1002877 (PMC3464226; doi:10.1371/journal.ppat.1002877)
Supplement: Table S1 — Suspected (extrapolated) exposure dates for 52 miners from the final Marburg hemorrhagic fever (MHF) patient list from the 1998–2000 outbreak in Durba, Democratic Republic of Congo. (DOCX) [file ppat.1002877.s001.docx]

Supplemental Table 1.

| **Occupation** | **Sex** | **Age** | **Date of Onset** | **Date of Death** | **Extrapolated Exposure Date** | **Exposure During High Risk Season** |
| --- | --- | --- | --- | --- | --- | --- |
| Miner | M | Ad* | Oct 98 | 20-Oct-98 | 13 Oct 1998 | N |
| Miner | M | 30 | 13-Nov-98 | 17-Nov-98 | 6 Nov 1998 | N |
| Miner | M | 20 | Jan 99 | --- | 26 Dec 1998 | Y |
| Miner | M | 23 | 7-Jan-99 | --- | 31 Dec 1998 | Y |
| Miner | M | 24 | 8-Jan-99 | 12-Jan-99 | 1 Jan 1999 | Y |
| Miner | M | 26 | 9-Jan-99 | --- | 2 Jan 1999 | Y |
| Miner | M | 24 | 15-Jan-99 | --- | 8 Jan 1999 | Y |
| Miner | M | 28 | 19-Jan-99 | 22-Jan-99 | 12 Jan 1999 | Y |
| Miner | M | 24 | 19-Jan-99 | 26-Jan-99 | 12 Jan 1999 | Y |
| Miner | M | 27 | Jan 99 | 27-Jan-99 | 13 Jan 1999 | Y |
| Miner | M | 35 | 20-Jan-99 | 25-Jan-99 | 13 Jan 1999 | Y |
| Miner | M | 32 | 25-Jan-99 | 31-Jan-99 | 18 Jan 1999 | Y |
| Miner | M | 56 | Jan 99 | 4-Feb-99 | 21 Jan 1999 | Y |
| Miner | M | 29 | 18-Dec-98 | 5-Feb-99 | 22 Jan 1999 | Y |
| Miner | M | 45 | 1-Feb-99 | 12-Feb-99 | 25 Jan 1999 | Y |
| Miner | M | 35 | 10-Feb-99 | 22-Feb-99 | 3 Feb 1999 | Y |
| Miner | M | 25 | 1-Jan-99 | 19-Feb-99 | 5 Feb 1999 | Y |
| Miner | M | 28 | 20-Feb-99 | 23-Feb-99 | 13 Feb 1999 | Y |
| Miner | M | 32 | 21-Feb-99 | 1-Mar-99 | 14 Feb 1999 | Y |
| Miner | M | 36 | 6-Mar-99 | 12-Mar-99 | 27 Feb 1999 | Y |
| Miner | M | 26 | 9-Mar-99 | 15-Mar-99 | 2 Mar 1999 | Y |
| Miner | M | 22 | 18-Mar-99 | 26-Mar-99 | 11 Mar 1999 | Y |
| Miner | M | 24 | Mar 99 | 27-Mar-99 | 13 Mar 1999 | Y |
| Miner | F | 24 | Mar 99 | 8-Apr-99 | 25 Mar 1999 | N |
| Miner | M | 36 | 2-Apr-99 | 13-Apr-99 | 26 Mar 1999 | N |
| Miner | M | 30 | 12-Apr-99 | 19-Apr-99 | 5 Apr 1999 | N |
| Miner | M | 20 | 18-Apr-99 | 22-Apr-99 | 11 Apr 1999 | N |
| Miner | M | 32 | 11-Jun-99 | --- | 4 Jun 1999 | N |
| Miner | M | 21 | 5-Jul-99 | --- | 28 Jun 1999 | Y |
| Miner | M | 34 | 1-Aug-99 | 15-Aug-99 | 26 Jul 1999 | Y |
| Miner | M | 19 | 6-Aug-99 | --- | 30 Jul 1999 | Y |
| Miner | M | 25 | 20-Dec-98 | 27-Dec-98 | 13 Dec 1999 | N |
| Miner | M | 41 | 26-Dec-99 | 26-Jan-00 | 19 Dec 1999 | Y |
| Miner | M | 24 | Dec 98 | 5-Jan-99 | 22 Dec 1999 | Y |
| Miner | M | Ad | 1-Jan-00 | 22-Jan-00 | 25 Dec 1999 | Y |
| Miner | M | 30 | 8-Jan-00 | 15-Jan-00 | 1 Jan 2000 | Y |
| Miner | M | 24 | 15-Jan-00 | 29-Jan-00 | 8 Jan 2000 | Y |
| Miner | M | 38 | 22-Jan-00 | 27-Jan-00 | 15 Jan 2000 | Y |
| Miner | M | 19 | 1-Feb-00 | Feb 00 | 25 Jan 2000 | Y |
| Miner | M | 28 | 11-Feb-00 | 18-Feb-00 | 4 Feb 2000 | Y |
| Miner | M | 22 | 13-Feb-00 | 23-Feb-00 | 6 Feb 2000 | Y |
| Miner | M | 31 | 23-Feb-00 | 1-Mar-00 | 16 Feb 2000 | Y |
| Miner | M | 23 | Feb 00 | 3-Mar-00 | 25 Feb 2000 | Y |
| Miner | M | 33 | 22-Apr-00 | 29-Apr-00 | 15 Apr 2000 | N |
| Miner | M | 19 | 3-Jul-00 | 13-Jul-00 | 26 Jun 2000 | Y |
| Miner | M | 25 | 10-Jul-00 | 13-Jul-00 | 3 Jul 2000 | Y |
| Miner | M | 27 | 19-Jul-00 | 26-Jul-00 | 12 Jul 2000 | Y |
| Miner | M | 30 | 5-Aug-00 | --- | 29 Jul 2000 | Y |
| Miner | M | 30 | 5-Aug-00 | 10-Aug-00 | 29 Jul 2000 | Y |
| Miner | M | 31 | 11-Aug-00 | 20-Aug-00 | 4 Aug 2000 | Y |
| Miner | M | 29 | 13-Aug-00 | 23-Aug-00 | 6 Aug 2000 | Y |
| Miner | M | Ad | 23-Aug-00 | 1-Sep-00 | 16 Aug 2000 | Y |

*Ad = adult

Suspected (extrapolated) exposure dates for 52 miners from the final Marburg hemorrhagic fever (MHF) patient list from the 1998-2000 outbreak in Durba, Democratic Republic of Congo (Pierre Rollin and Robert Swanepoel; personal communication on behalf of International Scientific and Technical Committee for Marburg hemorrhagic fever control in the Democratic Republic of Congo). All cases listed herein met the MHF case definition (Bausch et al., 2006) and are suspected of being primary exposures with no known previous contact with other MHF cases prior to disease onset. The date of exposure was extrapolated by subtracting seven days from the date of disease onset (median time from exposure to onset was previously estimated to be seven days, Bausch et al). The onset date, when not known, was extrapolated by subtracting seven days (Bausch et al.,) from the date of death. A “Y” in high risk exposure column indicates a suspected exposure date to have occurred during one of the proposed ‘high risk’ seasons. An “N” denotes those exposures to have occurred outside the ‘high risk’ seasons.
