# Supplementary material for: Seasonal Pulses of Marburg Virus Circulation in Juvenile Rousettus aegyptiacus Bats Coincide with Periods of Increased Risk of Human Infection
Source: PLoS Pathog. 2012 Oct 4;8(10):e1002877. doi: 10.1371/journal.ppat.1002877 (PMC3464226; doi:10.1371/journal.ppat.1002877)
Supplement: Table S2 — GenBank accession numbers of all Marburg virus sequences analyzed. (DOCX) [file ppat.1002877.s002.docx]

Table S2.

| **Sequence** | **NP-fragment** | **VP35-fragment** | **Whole genome** |
| --- | --- | --- | --- |
| 01Uga/USA 2007 | JX462492 | JX462503 |  |
| 549QBat Uga 2009 | JX462491 | JX462504 |  |
| 789QBat Uga 2009 | JX462493 |  |  |
| 846QBat Uga 2009 | JX462495 |  |  |
| 849QBat Uga 2009 | JX462502 |  |  |
| 960QBat Uga 2009 | JX462494 | JX462505 |  |
| 1079QBat Uga 2009 | JX462496 |  |  |
| 1232QBat Uga 2009 | JX462497 | JX462506 |  |
| 1261QBat Uga 2009 | JX462500 |  |  |
| 1368QBat Uga 2009 | JX462498 | JX462507 |  |
| 1431QBat Uga 2009 | JX462499 | JX462508 |  |
| 1511QBat Uga 2009 | JX462501 |  |  |
| 1304QBat Uga 2009 |  |  | JX458857 |
| 843QBatUga 2009 |  |  | JX458855 |
| 1175QBatUga 2009 |  |  | JX458854 |
| 53QBatUga 2008 |  |  | JX458852 |
| 914QBatUga 2009 |  |  | JX458856 |
| 164QBatUga 2008 |  |  | JX458853 |
| 1328QBatUga 2009 |  |  | JX458858 |
| 01Uga07 |  |  | FJ750957 |
| 02Uga07 |  |  | FJ750953 |
| 44Bat 2007 |  |  | FJ750954 |
| 188Bat 2007 |  |  | FJ750955 |
| 982Bat 2008 |  |  | FJ750956 |
| 331Bat 2007 |  |  | FJ750959 |
| 371Bat 2007 |  |  | FJ750958 |
| 782Bat 2007 | FJ743669 |  |  |
| 276Bat 2007 | FJ743670 | FJ743678 |  |
| 288Bat 2007 | FJ743672 | FJ743680 |  |
| 328Bat 2007 | FJ743674 | FJ743682 |  |
| 1013Bat 2008 | FJ743676 | FJ743684 |  |
| 772Bat 2007 | FJ743671 | FJ743679 |  |
| 291Bat 2007 | FJ743673 | FJ743681 |  |
| 427Bat 2007 | FJ743675 | FJ743683 |  |
| 883Bat 2008 | FJ743677 | FJ743685 |  |
| 01DRC 1999 |  |  | JX458833 |
| 02DRC 1999 |  |  | JX458851 |
| 03DRC 1999 |  |  | JX458826 |
| 04DRC 1999 |  |  | JX458825 |
| 05DRC 1999 |  |  | DQ447651 |
| 06DRC 1999 |  |  | JX458830 |
| 07DRC 1999 |  |  | DQ447650 |
| 09DRC 1999 |  |  | DQ447652 |
| 12DRC 2000 |  |  | JX458840 |
| 13DRC 2000 |  |  | JX458845 |
| 14DRC 2000 |  |  | JX458831 |
| 15DRC 2000 |  |  | JX458847 |
| 16DRC 2000 |  |  | JX458839 |
| 17DRC 2000 |  |  | JX458838 |
| 18DRC 2000 |  |  | JX458842 |
| 19DRC 2000 |  |  | JX458828 |
| 20DRC 2000 |  |  | JX458841 |
| 21DRC 2000 |  |  | JX458843 |
| 22DRC 2000 |  |  | JX458848 |
| 23DRC 2000 |  |  | JX458835 |
| 24DRC 2000 |  |  | JX458834 |
| 25DRC 2000 |  |  | JX458849 |
| 26DRC 2000 |  |  | JX458844 |
| 27DRC 2000 |  |  | JX458837 |
| 28DRC 2000 |  |  | JX458846 |
| 29DRC 2000 |  |  | JX458836 |
| 30DRC 2000 |  |  | JX458832 |
| 32DRC 2000 |  |  | JX458827 |
| 33DRC 2000 |  |  | JX458850 |
| 34DRC 2000 |  |  | JX458829 |
| 0215 Ang 2005 |  |  | DQ447658 |
| 0126 Ang 2005 |  |  | DQ447656 |
| 1386 Ang 2005 |  |  | DQ447655 |
| 1381 Ang 2005 |  |  | DQ447654 |
| 0181 Ang 2005 |  |  | DQ447653 |
| 1411 Ang 2005 |  |  | DQ447653 |
| 1380 Ang 2005 |  |  | DQ447653 |
| 1379c Ang 2005 |  |  | DQ447653 |
| 0754 Ang 2005 |  |  | DQ447659 |
| 0214 Ang 2005 |  |  | DQ447657 |
| 0998 Ang 2005 |  |  | DQ447660 |
| 2296 Gab 2006 | EU068110 | EU068113 |  |
| 1631 Gab 2005 | EU068109 | EU068112 |  |
| 1448 Gab 2005 | EU068108 | EU068111 |  |
| Ozo Zim 1975 |  |  | AY358025 |
| Mus Ken 1980 |  |  | DQ217792 |
| Rav Ken 1987 |  |  | DQ447649 |
| Pop Uga/Ger 1967 |  |  | Z29337 |
| 01 Uga/Net 2008 |  |  | JN408064 |
